# Bacteriophage-insensitive mutants for high quality Crescenza manufacture

**DOI:** 10.3389/fmicb.2014.00201

**Published:** 2014-05-06

**Authors:** Donatella Chirico, Arianna Gorla, Viola Verga, Per D. Pedersen, Eliseo Polgatti, Antonio Cava, Fabio Dal Bello

**Affiliations:** Clerici–Sacco Group, Caglificio Clerici S.p.A. and Sacco S.r.l., CadoragoItaly

**Keywords:** *Streptococcus thermophilus*, bacteriophages, bacteriophage-insensitive mutants, dairy fermentation, cheese, starter culture

## Abstract

*Streptococcus thermophilus* is a thermophilic lactic acid bacterium used as starter culture for the manufacture of fermented dairy products. For the production of Crescenza and other soft cheeses, Sacco has developed and provides dairies with three different defined blends of *S. thermophilus* strains. Each blend contains two different *S. thermophilus* strains. The strains were selected based on their unique technological properties as well as different phage profiles. Analysis of 133 whey samples collected in 2009–2010 from Italian dairies showed a high prevalence (about 50%) of bacteriophage attacks on the blend ST020. More specifically, the strain *S. thermophilus* ST1A was found to be the preferred target of the bacteriophages. A bacteriophage insensitive mutant (BIM5) of the phage-sensitive strain ST1A was successfully developed and used to substitute strain ST1A in the Crescenza starter culture ST020. The strain BIM5 showed identical technological and industrial traits as those of the phage-sensitive strain ST1A. The improved resistance of the modified Crescenza starter culture ST020R was confirmed at Italian dairies, and its effectiveness monitored on 122 whey samples collected in 2011–2012. Compared to the previous values (2009–2010), the use of the phage-hardened blend ST020R allowed reducing of frequency of phage attacks from about 50 to less than 5% of the whey samples investigated.

## INTRODUCTION

**FIGURE 1:**
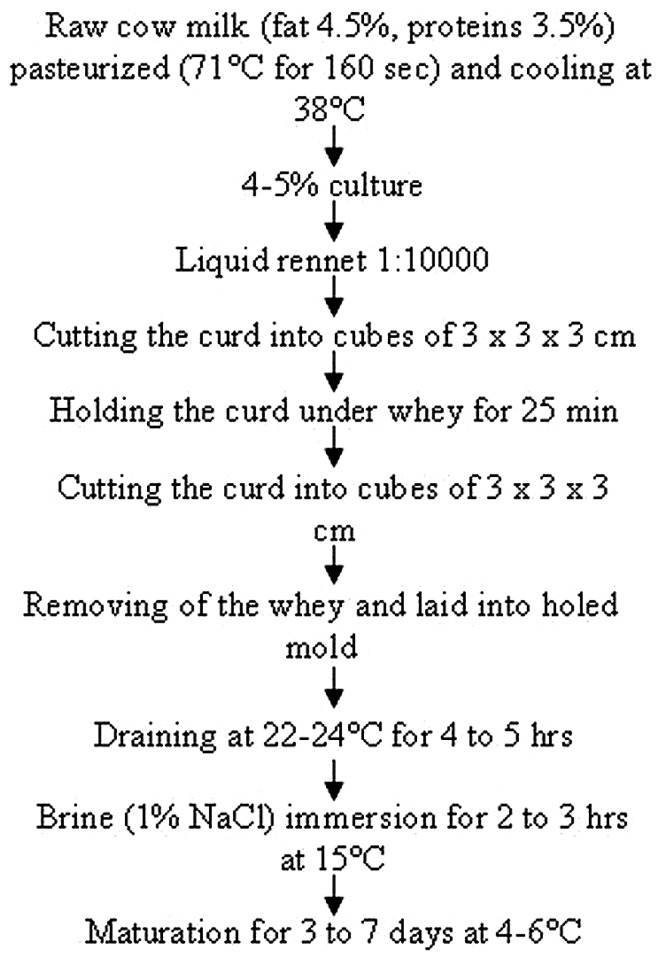
**Schematic representation of the Crescenza manufacturing process**.

*Streptococcus thermophilus* is the second most important industrial species after *Lactococcus lactis* (Fox and Cogan, 1993; Hols et al., 2005) and it is used in the manufacture of yogurt as well as many traditional Italian cheeses such as Mozzarella and Crescenza. In particular, Crescenza is a member of the “stracchino” cheeses family, but differs from the others since it is consumed fresh, without a ripening of about 20 days that is used for other members of the family. A schematic overview of the Crescenza manufacturing process is presented in **Figure 1**. Crescenza typical taste is milky, sweetish and mild, its paste is homogeneous and rather compact, pure white and buttery, melting in the mouth. The manufacture of Crescenza – and of cheese in general – requires the inoculation of milk with carefully selected bacterial strains blends, known as starter cultures, allowing the control of fermentation and production of high-quality fermented products. For the production of Crescenza, Sacco provides three blends of starter cultures each composed by two strains of *S. thermophilus*. Typically, the *S. thermophilus* strains are selected based on peculiar technological characteristics, such as low proteolytic (PrtS^-^) and reduced mesophilic activities, as well as the ability to give the typical milky taste after fermentation.

Because significant amounts of bacterial cells are cultivated in the fermentation vats, most cheese industries have experienced problems with phage contaminations (Emond and Moineau, 2007). The first description of phages affecting a dairy starter culture was reported by Whitehead and Cox (1936), and even if since then the field has seen significant improvements, all over the world bacteriophages are still the major cause of dairy fermentations failures. Although raw milk is one of the source of phages in the industrial environment (Bruttin et al., 1997; Moineau and Lévesque, 2005), several dispersion pathways may occur in dairies. Personnel movements or transport of equipment and/or raw materials might cause the dispersion of phage particles as an aerosol (Verreault et al., 2011). In addition, phages present in recycled by-products may also spread to the entire factory environment, since they can remain in the air for long periods (Hinrichs, 2001). The first solution has been the alternate use (i.e., rotations) of starter cultures composed by strains that have different phage profiles (Thunell et al., 1985). In spite of this, phages are still causing significant losses in dairies and for this reason many studies are focusing on understanding phage-host dynamics, with special attention to bacterial resistance mechanisms (Allison and Klaenhammer, 1998; Labrie et al., 2010). The generation of Bacteriophage-Insensitive Mutants (BIMs), described by Mills et al. (2007), has received particular attention by starter culture manufacturers. It describes a relatively simple protocol that can be applied in any laboratory and that allows the generation of mutations in the sensitive microorganisms, that confer resistance to attacking phages. Increased resistance is typically achieved through mutations affecting bacterial-surface molecules interfering especially with phage attachment. BIMs are useful to fermentation-based industries; in fact it is possible to introduce them in the starter culture instead of the strains, which were sensible to a phage or a large number of phages, and this strategy increases the rotations robustness (Coffey and Ross, 2002).

Our phage analysis laboratory routinely investigate whey samples from dairy manufacturers, in order to detect the presence of phages, monitor performance of specific strains/starter cultures, as well as isolate new/novel bacteriophages. In particular, the whey samples of several Italian dairies producing Crescenza were controlled for 2 years, and the analysis of these results indicated that one starter culture, and particularly one *S. thermophilus* strain was showing high percentages of phage attacks. In this study, we have applied the BIM protocol described by Mills et al. (2007) to increase the phage resistance of the most sensitive *S. thermophilus* strains present in a Crescenza starter culture. The resulting mutant was used to generate a more robust starter culture, and its effectiveness was monitored over the following 2 years of use at Italian Crescenza manufacturers.

## MATERIALS AND METHODS

### CRESCENZA STARTER CULTURES, STRAINS AND CULTURE CONDITIONS

For Crescenza manufacture, Sacco produces 3 blends of PrtS^-^ *S. thermophilus*, namely ST020, ST022 and ST024, each composed by 2 strains. In particular, the blend ST020 contains the strains ST1A and ST1B, the blend ST022 contains the strains ST2C and ST2D, and the blend ST024 contains the strains ST3E and ST3F. The BIMs generated from the strain ST1A were identified as BIM1, BIM2, BIM3, BIM4, BIM5, and BIM6.

*Streptococcus thermophilus* strains were routinely grown in 10% (w/v) skim milk powder (Irish dairy board, Dublin, Ireland) containing 0.1% (w/v) yeast extract (BD, Difco lab, Sparks, USA; LPEL broth) and heat-treated at 115°C for 15 min (LE). Frozen vials of each strain were inoculated at 2% (v/v) in 10 ml LPEL and incubated at 42°C until clotting. From this passage, the cell count (CFU/ml) was determined by serial dilutions on MRS agar (Oxoid, Hampshire, England) as described in the ISO method 27205 (2010).

### pH-MULTISCAN ANALYSIS

Whey samples were collected from local dairies accounting for a total of 133 and 122, in the periods 2009–2010 and 2011–2012, respectively. Wheys were checked for phage content using a pH-Multiscan according to the manufacturer instructions (HNH Consult APS, Denmark). Briefly, 96-wells microtiter plates is inoculated with LE containing the two pH indicators bromocresol green and bromocresol purple. For each strain two wells are used, one containing 300 μl of LE and inoculated only with the strain under investigation (LES), and the second well containing 300 μl of LE inoculated with the strain as well as 0.3% of previously filtered (0.45 μm) whey (LEW). The analysis is performed in triplicate. The plates are incubated at 37°C, a scanner monitors the color (pH values) every minute, and a software translate the recorded values into acidification curves. A delay in the acidification curve of the LEW well compared to the LES well, indicates presence in the whey sample of phage/s attacking the inoculated strain.

### BACTERIOPHAGES, PREPARATION AND ENUMERATION

The two *S. thermophilus* bacteriophages φ118 and φ215 were used to generate BIMs of the strain ST1A. The phages were considered different based on their host range, i.e., each of the phages attacks different *S. thermophilus* strains. Seventy-eight *S. thermophilus* phages of Sacco phage collection were used to check the phage profile of the mutants. These phages were isolated over the last 20 years from whey samples collected from different dairies mainly from Europe, South America and Asia.

Bacteriophage enumeration was achieved as follows. The strain ST1A was inoculated at 2% in 10 ml LE and 1% of the phages φ118 or φ215 were added. A control culture, without phages, was also prepared. Tubes were incubated at 42°C until clotting of the control. The samples with phages, which remained liquid, were added with 0.5% of lactic acid (sterilized at 121°C for 15 min), centrifuged at 4000 rpm for 15 min and then filtered through a 0.45 μm sterile filter. Bacteriophages were enumerated using the plaque test assay technique as described by Lillehaug (1997).

### GENERATION OF BIMs AND DEVELOPMENT OF A NEW RESISTANT BLEND

Bacteriophage insensitive muntants of strain STA1 were generated and characterized by the 3-step process as described by Mills et al. (2007), using a multiplicity of infection of 10. The stability of the BIMs was controlled by growing each mutant in LE alone at 42°C for 10 passages (corresponding to circa 60 generations), and challenging the mutant with the phages φ118 and φ215. The colony morphology after the 10th passage was also compared to that of ST1A by streaking out on HHD agar as described by Camaschella et al. (1998). Acidification tests were performed in 9% (w/v) skim milk powder by continuous pH measurements with Cinac4 (Alliance Instruments Dairy Division, France) as described in the ISO method 26323 (2009). Acidification data of the BIMs were compared with those of the strain ST1A. The resistance of the BIMs against 80 *S. thermophilus* phages was investigated using the pH-Multiscan as described above. The generated BIMs were also compared to the parental strain, by using Pulsed Field Gel Electrophoresis (PFGE), using the restriction enzyme SmaI as described by Mills et al. (2007). The BIMs more similar to the strain ST1A were selected for pilot scale production, blended at 50% ratio with the strain ST1B, and the acidification performance of the resulting starter culture ST020R was compared to that of ST020 using the Cinac4 as described above.

### TECHNOLOGICAL TEST OF THE NEW BLEND

To verify if the blend ST020R containing the BIM of ST1A was suitable for the production of high quality Crescenza, at a pilot plant of a local dairy two Crescenza productions were performed, one with ST020 and one with ST020R. Briefly, 2000 L of cow milk (4.5% fat and 3.5% proteins) were heat-treated at 71°C for 160 s and then cooled to 38°C. The starter culture and liquid rennet 1:10000 (Caglificio Clerici, Cadorago, Italy) were added and incubated at 38°C for 30 min. The curd was broken in two phases into cubes of 3 cm × 3 cm × 3 cm, with a rest of ca 25 min in between. The mass was mixed again, the whey was removed, laid in holed mold, i.e., square plastic boxes with sides of 20 cm, and stewed for 4–5 h at 22–24°C. The curd was performed by immersing the cheese in brine (1% NaCl) for 2–3 h at 14–16°C. The cheese was matured for 3–7 days. The Crescenza produced with either starter culture was tasted by a professional team of sensorial evaluators.

## RESULTS

### ANALYSIS OF WHEYS AND IDENTIFICATION OF THE STRAIN TO BE HARDENED

**FIGURE 2:**
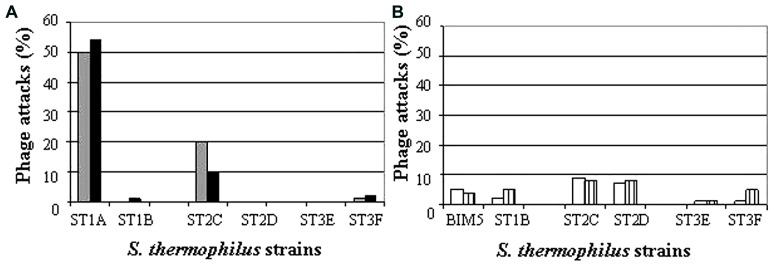
**Frequency of phage attacks of the strains contained in the standard Crescenza blends ST020 (ST1A and ST1B), ST022 (ST2C and ST2D), and ST024 (ST3E and ST3F) as well as the phage-hardened blend ST020R (BIM5 and ST1B).** Each strain was grown in milk or in milk with whey sample. A delay in growth rate of a strain by the addition of whey was considered as a phage attack. **(A)**, whey samples collected during the years 2009 (grey) and 2010 (black); **(B)**, whey samples collected during the years 2011 (white) and 2012 (vertical line pattern).

Routinely, whey samples from dairy customers are analyzed with the method described in section “pH-Multiscan Analysis.” All the data collected are used to monitor efficacy of the starter cultures used, which is expressed as frequency of whey samples containing phages able to attack the strains present in the starter cultures. Concerning the blends ST020, ST022, and ST024 sold for Crescenza manufacture, the results of attacks on single strains contained in each blend are summarized in **Figure 2A**. In particular, the analysis of 68 and 65 samples collected in 2009 and 2010, respectively, showed high frequencies of attacks for the blend ST020, and more specifically for the strain ST1A. The second most attacked strain was ST2C, used in ST022. Strain ST1A was therefore selected to be hardened against the two phages φ118 and φ218 isolated from whey samples, and previously inserted in Sacco phage collection.

### GENERATION AND CHARACTERIZATION OF BIMs

**FIGURE 3:**
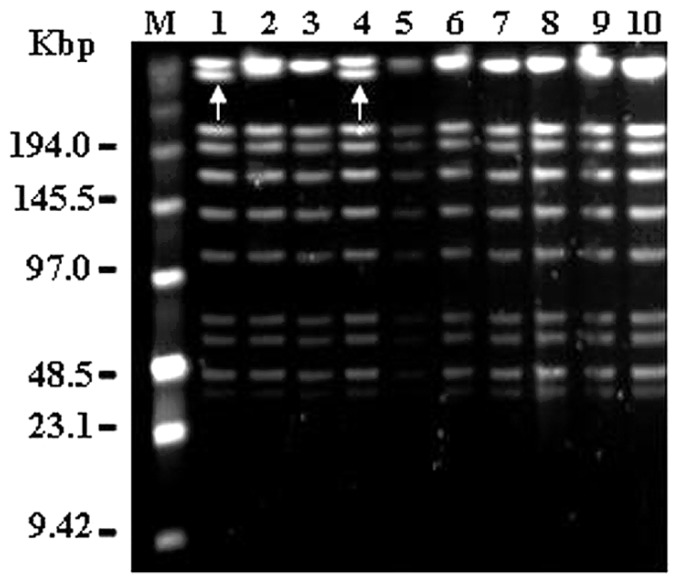
**Pulsed Field Gel Electrophoresis profiles of the phage-sensitive strain ST1A and its generated BIMs.** The enzyme used for digestion is SmaI. ST1A, lane 1 and 4; BIM1, lane 2; BIM2, lane 3; BIM3, lane 5; BIM4, lane 6; BIM5, lane 7; BIM6, lane 8; BIM7, lane 9; BIM8, lane 10. The white arrow indicates the band that is missing in the BIMs profiles.

Eight BIMs (BIM1 to BIM8) of ST1A were generated using the 3-step process described by Mills et al. (2007). The BIMs and ST1A were subjected to PFGE analysis, stability and acidification tests as well as phage profile by using the 80 *S. thermophilus* phages available. The results of the performed test are summarized in **Table 1**. PFGE analysis of the DNA isolated from the 8 BIMs revealed an almost identical profile to that of ST1A (**Figure 3**), apart from the lack of one band (white arrow, **Figure 3**). To determine stability of the acquired phage-resistance, the BIMs were grown for about 60 generations without selective pressure, i.e., presence of phages. When exposed to the presence of the phages φ118 and φ218, only six out of the eight generated BIMs maintained their resistant profile, and were thus subjected to the acidification test (**Table 1**). Comparing the acidification performance of the six stable BIMs to that of the phage-sensitive strain ST1A revealed that only BIM1, BIM4, BIM5, and BIM6 maintained an acidification activity similar to that of ST1A (data not shown), and they were thus selected for further analysis. The four selected BIMs were controlled against further 80 phages, and only two of them, namely BIM4 and BIM5, were found resistant to all investigated phages (**Table 1**). These two BIMs were produced at the pilot plant, and used for further tests.

**Table 1:** Genetic and physiological tests performed on the eight BIMs generated from the phage-sensitive strain ST1A.

	Analysis type			
BIM code	PFGE profile	Stability^a^	Acidification	Phage profile against 78 phages^b^
BIM1	Similar to ST1A	Stable	Similar to ST1A	Sensitive^c^
BIM2	Similar to ST1A	Stable	Slow	n.d.
BIM3	Similar to ST1A	Stable	Slow	n.d.
BIM4	Similar to ST1A	Stable	Similar to ST1A	Resistant^d^
BIM5	Similar to ST1A	Stable	Similar to ST1A	Resistant
BIM6	Similar to ST1A	Stable	Similar to ST1A	Sensitive
BIM7	Similar to ST1A	Instable	n.d.	n.d.
BIM8	Similar to ST1A	Instable	n.d.	n.d.

a Assessed by growing the BIMs without phages for ca. 60 generations and thereafter exposing cells to the phages ϕ118 and ϕ218.

b All phages from the phage collection were used to assess the sensitivity of the BIMs.

c Attacked by at least 1 phage out of the 78 tested.

d Resistant to all 78 phages.

### LABORATORY FORMULATION OF A CRESCENZA STARTER CULTURE CONTAINING A BIM

**FIGURE 4:**
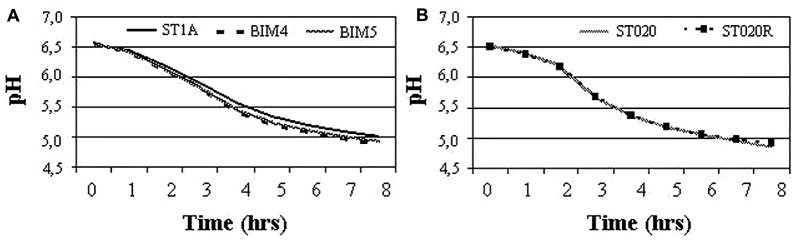
**Acidification profiles of the phage-resistant strains BIM4 and BIM5 produced at the pilot plant as frozen pellets, compared to that of frozen pellets of the phage-sensitive strain ST1A (A).** Acidification performance of the blend ST020R containing the BIM5, compared to that of the phage-sensitive blend ST020 **(B)**.

When produced at the pilot plant, BIM4 and BIM5 showed comparable growth rate and overall yield to that of the strain ST1A (data not shown). Both mutants were produced as frozen pellets, and their acidification performance was investigated as described above. The data collected revealed an acidification profile similar to that of the parental strain ST1A (**Figure 4A**). For simplicity, the strain BIM5 was selected and used to substitute the strain ST1A in the blend ST020, giving rise to the new blend ST020R.

Using the acidification protocol described in paragraph 2.5, the performance of the phage-hardened blend ST020R was found identical to that of the phage-sensitive blend ST020 (**Figure 4B**).

### CRESCENZA MANUFACTURE WITH THE PHAGE-HARDENED STARTER CULTURE ST020R

Having proven that the performance of the phage-hardened starter culture ST020R was identical to that of ST020 under laboratory conditions, the next step was to prove its suitability for production of high quality Crescenza at a local Crescenza manufacturer. To reduce the variability due to different lots of cheese milk, on the same day 2000 L of cheese milk was fermented either with ST020 (control) or with the phage-hardened blend ST020R. During production no significant difference was observed when using the blend ST020R (data not shown), and also a sensory evaluation throughout the shelf life of the Crescenza produced did not reveal significant differences between the Crescenza produced with ST020 or ST020R starter cultures. The new blend ST020R was used at the same inoculation level as the blend ST020, and it did not contain a higher *S. thermophilus* cell count (data not shown).

### CONTINUOUS MONITORING OF THE PHAGE-HARDENED BLEND EFFICACY

The improved blend ST020R substituted the phage-sensitive blend ST020. Routine monitoring of the whey samples arriving from different dairies continued over the years 2011 and 2012. The percentage of whey samples analyzed and found to contain phages able to attack the strains present in the 3 Crescenza starter cultures was measured as shown in **Figure 2B**. The analysis of 64 and 58 whey samples collected in 2011 and 2012, respectively, showed a low percentage of attacks in particular for the strains present in ST020R. In particular, the BIM5 clearly revealed a lower percentage of phage attacks when compared to the values recorded for ST1A, allowing to reduce the frequency of phage attacks from ca. 50% for the blend ST020 in the years 2009–2010 to less than 5% for the blend ST020R in the years 2011–2012. The new blend ST020R was used at the same inoculation level of the blend ST020, and it did not contain a higher *S. thermophilus* cell count (data not shown).

## DISCUSSIONS

Virulent lactic acid bacteria phages are the cause of serious problems during manufactures of dairy products, and industries are constantly waging war against these viruses to keep them under control. In the Italian productions of cheese and yogurt, phage infections of *S. thermophilus* are becoming a more persistent problem. While supplying 3 starter cultures to Crescenza manufacturers, we observed over a 2 years period that the strain ST1A contained in the blend ST020 was frequently attacked by phages (**Figure 2A**). Our results were confirmed by the cheese manufacturers, who experienced several production problems when using this particular blend in the period under investigation (data not shown). By using the BIM protocol described by Mills et al. (2007) we phage-hardened the strain ST1A, and selected the BIM5 as a substitute of the parental strain in the blend ST020. The use of the resulting phage-hardened blend ST020R allowed to significantly reduce the frequency of phage attacks (circa 10-fold) when monitoring whey samples collected over the following 2 years (**Figure 2B**).

The identification of a phage sensitive strain within starter cultures is a common experience for starter culture manufacturers, and it is often linked to the frequency of use of the strain/blend at a particular dairy. Phage collections are commonly used to characterize and differentiate strains that are included in culture collections, and thus one simple solution would seem the replacement of a phage-sensitive strain with a phage-insensitive from the culture collection. However, many important technological traits are strain-specific and therefore the replacement is often not possible or desirable (Capra et al., 2011). Furthermore, the industrialization of a novel strain requires high investments, due to scaling up and optimization of fermentation conditions, and there is no assurance that the strain selected as replacement will be resistant to phages present in the dairies. To overcome this problem, Mills et al. (2007) published an efficient and low-cost three step method of isolating BIMs of *S. thermophilus* by multiple passages in milk in the presence of phages against which protection is required. There are many other published methods for spontaneous BIM generation. Generally, they involve the challenge of cultures with high levels of single (Coffey et al., 1998; Guglielmotti et al., 2009) or multiple (Marcó et al., 2011a,b) bacteriophages, and they have different degrees of success depending on the species and strain considered. BIMs of the original strain may be isolated which are resistant to the offending phage while retaining their technologically important attributes. For this reason, when applying Mills et al. (2007) protocol we have included several control steps of the generated BIMs (**Table 1**), to assure that no major genetic reassembly occurred (**Figure 3**), that the growth in milk and that the performance of the starter culture was still maintained both under laboratory conditions (**Figure 4B**) as well during Crescenza manufacture. Of the eight BIMs obtained, only two maintained the desirable traits of the strain ST1A. The loss of stability of the phage-resistant phenotype that we observed for BIM7 and BIM8 has already been observed (Mahony et al., 2012). Changes in cell wall polysaccharides structure or mutations in key biosynthetic genes are expected to cause detrimental effects on growth and/or cell division, which might explain the reduced growth phenotype we observed for BIM2 and BIM3 when they were inoculated in laboratory milk (data not shown). Remarkably, BIM1 and BIM6 showed to have become sensitive against other phages present in the phage collection, thus underlining the importance of controlling that all desirable traits of the phage-sensitive strain are maintained in the generated BIMs.

Unfortunately, bacteriophages rapidly evolve (due to high frequency of mutations) to overcome any changes in the host receptor and/or resistance mechanisms of their target strains. Analysis of phage mutants with expanded/altered host ranges demonstrates that point mutations in structural tail genes is the sole requirement to overcome changes in host receptors, improve host adsorption or to infect new strains (Ravin et al., 2002; Duplessis et al., 2006; Uchiyama et al., 2011). As such mutations can rapidly occur, there is no indication and assurance concerning the duration of the robustness of a particular BIM/starter culture in the dairies. In the industrial praxis, some BIMs have shown to confer resistance for several years, as we have clearly described in this work, whereas other failed within few months of applications (own observations, data not shown). Understanding the evolutionary and molecular processes that allow phages to overcome host barriers may provide novel indications for the development of the next generation of phage-resistant starter cultures. For the time being, starter culture manufacturers will be daily challenged to produce and provide high quality starter cultures that can compete and resist a quickly evolving bacteriophage community. Accurate strain selection and starter culture development, correct use of starter culture rotations as well as strict hygiene conditions in the dairies will still remain the key steps for the successful production of high quality dairy products such as Crescenza.

## Conflict of Interest Statement

The authors declare that the research was conducted in the absence of any commercial or financial relationships that could be construed as a potential conflict of interest.
